# Melflufen - a peptidase-potentiated alkylating agent in clinical trials

**DOI:** 10.18632/oncotarget.18420

**Published:** 2017-06-08

**Authors:** Malin Wickström, Peter Nygren, Rolf Larsson, Johan Harmenberg, Jakob Lindberg, Per Sjöberg, Markus Jerling, Fredrik Lehmann, Paul Richardson, Kenneth Anderson, Dharminder Chauhan, Joachim Gullbo

**Affiliations:** ^1^ Department of Medical Sciences, Division of Clinical Pharmacology, Uppsala University, Uppsala SE, Sweden; ^2^ Department of Women’s and Children’s Health, Childhood Cancer Research Unit, Karolinska Institutet, Stockholm, Sweden; ^3^ Department of Immunology, Genetics and Pathology, Uppsala University, SE-75185, Uppsala, Sweden; ^4^ Oncopeptides AB, Västra Trädgårdsgatan 15, Stockholm, Sweden; ^5^ Recipharm OT Chemistry AB, Sweden; ^6^ Department of Medical Oncology, The LeBow Institute for Myeloma Therapeutics and Jerome Lipper Myeloma Center, Dana-Farber Cancer Institute, Harvard Medical School, Boston, Massachusetts

**Keywords:** melflufen, aminopeptidase, cancer, targeted chemotherapy

## Abstract

Aminopeptidases like aminopeptidase N (APN, also known as CD13) play an important role not only in normal cellular functioning but also in the development of cancer, including processes like tumor cell invasion, differentiation, proliferation, apoptosis, motility, and angiogenesis. An increased expression of APN has been described in several types of human malignancies, especially those characterized by fast-growing and aggressive phenotypes, suggesting APN as a potential therapeutic target.

Melphalan flufenamide ethyl ester (melflufen, previously denoted J1) is a peptidase-potentiated alkylating agent. Melflufen readily penetrates membranes and an equilibrium is rapidly achieved, followed by enzymatic cleavage in aminopeptidase positive cells, which results in trapping of less lipophilic metabolites. This targeting effect results in very high intracellular concentrations of its metabolite melphalan and subsequent apoptotic cell death. This results in a potency increase (melflufen *vs* melphalan) ranging from 10- to several 100-fold in different *in vitro* models. Melflufen triggers a rapid, robust, and an irreversible DNA damage which may account for its ability to overcome melphalan-resistance in multiple myeloma cells. Furthermore, anti-angiogenic properties of melflufen have been described.

Consequently, it is hypothesized that melflufen could provide better efficacy but no more toxicity than what is achieved with melphalan, an assumption so far supported by experiences from hollow fiber and xenograft studies in rodents as well as by clinical data from patients with solid tumors and multiple myeloma. This review summarizes the current preclinical and clinical knowledge of melflufen.

## INTRODUCTION

An increased expression of various hydrolytic enzymes like peptidases, esterases, and proteases has been described in several types of human malignancies, especially those characterized by fast-growing and aggressive phenotypes [1]. The Zn^2+^-dependent membrane-bound ectopeptidase aminopeptidase N (APN, also known as CD13), widely expressed in mammalian cells, plays an important role in the development of cancer, including processes like tumor cell invasion, differentiation, proliferation, apoptosis, motility, and angiogenesis [2-11]. The multiple functions of APN have lead to its designation as a “moonlighting ectoenzyme” [12]. Together, these abilities suggest APN as a potential therapeutic target in the treatment of cancer. Different approaches have been used to develop new drugs directed at this target, including enzyme inhibitors and APN-targeted carrier constructs, as reviewed [10]. Several APN-directed therapies have been investigated clinically, for example, the inhibitor Ubenimex (bestatin) [10].

**Figure 1:**
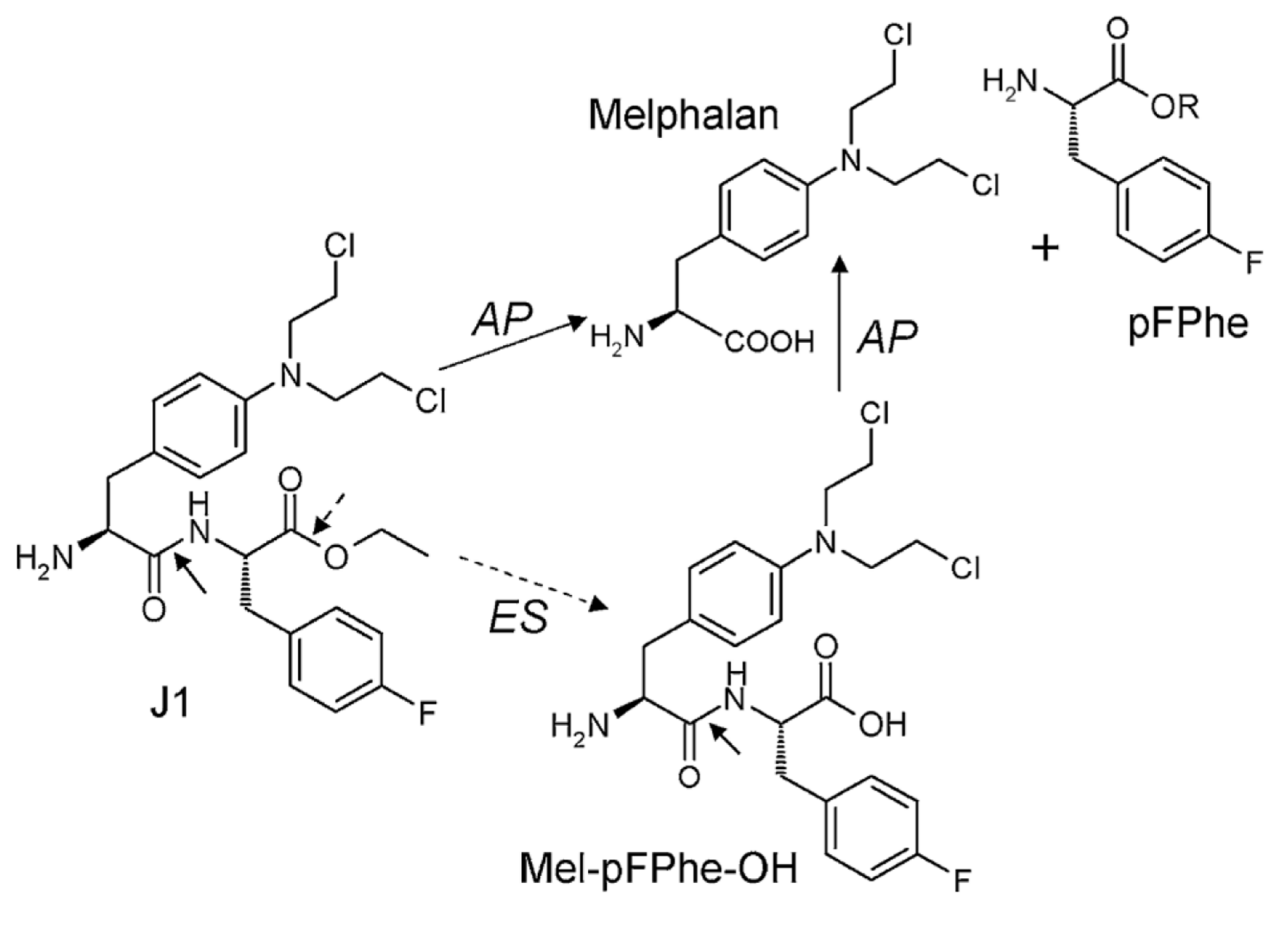
Chemical structure of melflufen (J1), melphalan, and the proposed targeting of tumor cells *via* APN-mediated cleavage Reprinted from [19] with permission. AP: Aminopeptidase; ES: Esterase.

Melphalan is a widely used classical chemotherapeutic agent in the group of alkylating agents that was developed more than 50 years ago, and substantial clinical experience has been accumulated [13, 14]. The drug has now been replaced by modern chemotherapeutics in most diagnoses, and currently melphalan treatment is in most countries limited to multiple myeloma and as a component of high-dose myeloablative regimens. The amino acid-based chemical structure of melphalan provides possibilities for modification of the *N*- and *C*-termini and incorporation into peptides, targeting its cytotoxicity to cells with peptide receptors and/or enzymatic activating systems (reviewed in [15]). One such derivative is melphalan flufenamide (L-melphalanyl-p-L-fluorophenylalanine ethyl ester hydrochloride), abbreviated melflufen and previously denoted J1 (Figure 1). By employment of a simple peptide bond, the activity of melflufen is directed to APN-expressing cells, providing a peptidase-potentiated effect.

This review summarizes the current preclinical and clinical knowledge of melflufen. Various *in vitro* and *in vivo* studies using different cancer models have consistently shown significantly higher activity of melflufen *versus* melphalan. The lipophilic characteristics of melflufen allow for a faster cellular uptake and its rapid cleavage into melphalan (and *p*-fluorophenylalanine) intracellularly. This peptide hydrolysis is mediated by aminopeptidases (like aminopeptidase N), allowing for a potentiated effect in APN-rich environments, resulting in accumulation of alkylating moieties in cancer cells, as repeatedly demonstrated in preclinical models. The above observations suggest that melflufen may provide better efficacy, but no more toxicity, than what is achieved with melphalan. Currently, melflufen is being evaluated in clinical trials in relapsed and refractory multiple myeloma.

## AMINOPEPTIDASE-POTENTIATED ACTIVITY

Chemically, melflufen may be described as the ethyl ester of a dipeptide consisting of melphalan and *para*-fluoro-L-phenylalanine. In a series of *in vitro* experiments, as detailed below, melflufen compared favorably with melphalan [16]. A subsequent structure-activity-relationship (SAR) analysis of melflufen and a series of other melphalan-containing dipeptide derivatives was performed in a panel of cell lines. Factors like amino acid composition and sequence, and modifications of the *C*- and *N*-termini of the dipeptide derivatives appeared to have strong influence on the *in vitro* activity, as well as, to a minor extent, the lipophilicity of the peptide. It should be noted that all tested peptide derivatives were substantially more lipophilic (melflufen’s estimated logP is 4) than melphalan. These results indicate that the activity of these compounds relies not only on their chemical reactivity but also on active biological interactions such as transport across membranes and/or enzymatic liberation of reactive molecular entities [17].

While an active transport mechanism has not yet been established, the enzymatic potentiation of the alkylating peptide’s cytotoxic activity was confirmed using peptidase inhibitors and analysis of dipeptide derivatives designed to resist the action of peptidases [18]. These studies demonstrated a rapid intracellular release of the alkylating moiety (i.e. free melphalan) in cells with high enzymatic activity. Specifically, a maximum intracellular melphalan concentration following melflufen exposure was reached within 15 min, which exceeded by more than 10-fold those concentrations achieved after an equimolar melphalan exposure [18]. Conversely, the aminopeptidase inhibitor bestatin blocked this intracellular accumulation and associated toxicity [18]. This rapid intracellular accumulation is dependent on a rapid transport of melflufen over the cell membrane, most probably by passive diffusion of the lipophilic molecule, driven by an enzymatic clearance of melflufen (i.e. formation of melphalan) in cells with high APN expression. As a consequence, melflufen´s activity is less dependent on exposure time than other chemotherapeutics, and almost full activity is obtained after 30 minutes of exposure *in vitro* [18]. This finding is important for the clinical situation with rather short half-life of melflufen in humans (see below).

The importance of aminopeptidases like APN for the potentiation of melflufen cytotoxicity has been described in several cell types [19], including multiple myeloma [20], as described below.

## PHARMACOKINETICS-PHARMACODYNAMICS *IN VITRO* AND *IN VIVO*

The APN-mediated cleavage of melflufen is an efficient and quick process, driven by the lipophilicity of the drug (logP 4.04) and the enzymatic release of melphalan and intracellular trapping [18, 19, 21]. This efficient intracellular delivery could be suppressed in both magnitude and time by the previously mentioned aminopeptidase inhibitor bestatin, and also by ebelactone A, an esterase inhibitor [19]. As expected, this suppression of hydrolysis also resulted in reduced cytotoxic effects of melflufen. *In vitro* assays with purified APN enzyme provided evidence for a specific role of APN in the hydrolysis of melflufen, which allowed for the release of free melphalan intracellularly [19]. Involvement of APN in melflufen-mediated cytotoxic and apoptotic signaling was also confirmed by using plasmid-based overexpression of APN or knockdown of endogenous APN with siRNA in different tumor cell lines, including multiple myeloma [19, 20]. Clearly, the role of APN in the activation of melflufen, together with its association with and overexpression in various tumors, suggests that melflufen is activated in a tumor selective manner [19].

**Figure 2:**
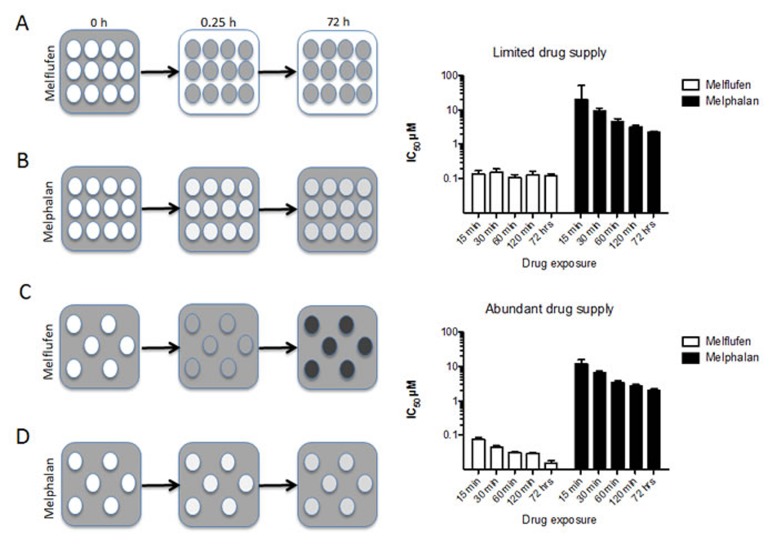
Schematic presentation of the time course for melflufen’s accumulation and cytotoxic effect **2A.** When cells are exposed to melflufen, the alkylating moieties are rapidly accumulated within the cells. With many cells, melflufen is rapidly consumed, and the difference between short and long exposures for the drug regarding cytotoxic activity (measured as cell death after 72 h incubation in drug-free medium) is modest (upper bar graph). In contrast, melphalan enters the cells slowly and reaches equilibrium over time **2B.**, resulting in higher activity with prolonged exposure time (lower bar graph). If the number of cells is limited, and melflufen supply abundant, a higher number of intracellular alkylating moieties per cell following melflufen is formed **2C.**, and as a result, cultures with lower cell densities are more sensitive than cultures with higher cell densities (bar graphs). This effect is becomes even more pronounced for long exposure times. In the absence of an active accumulating mechanism the cell density has little or no influence on melphalan’s drug accumulation **2B.**, **2D.** or activity (lower bar graphs). These data are modified from [23].

This very rapid accumulation of melphalan (intracellular Cmax of melphalan obtained within 15 minutes [18, 19]) in cells has two very important implications. First of all, the APN-driven competition for melflufen results in very high melphalan concentrations preferentially in cells with high APN expression [19], and tumor cells (shown only *in vitro*) are loaded with efficient amounts of melphalan within less than an half-an-hour of exposure [18]. Secondly, this load of active alkylating moieties in tumor cells triggers rapid, robust, and irreversible DNA damage, which may account for melflufen’s ability to overcome melphalan resistance [22]. The principles for intracellular trapping and competitive accumulation are summarized in Figure 2 (HL60 AML cell line data modified from [23]). When melflufen is present in limited amounts per cell (i.e. high cell densities) the drug is rapidly absorbed by the cells (in a competitive manner) and prolonged exposure does not affect the IC_50_ (Figure 2A and top bar graph). When melflufen is present in excess (i.e. low cell densities), prolonged exposure decreases the IC_50_-values (Figure 2C and lower bar graph). For melphalan, that enters the cells considerably slower, the cell density has little effect on IC_50_, which is numerically similar in low and high cell densities, and prolonged exposure is consistently associated with a better effect.

**Figure 3:**
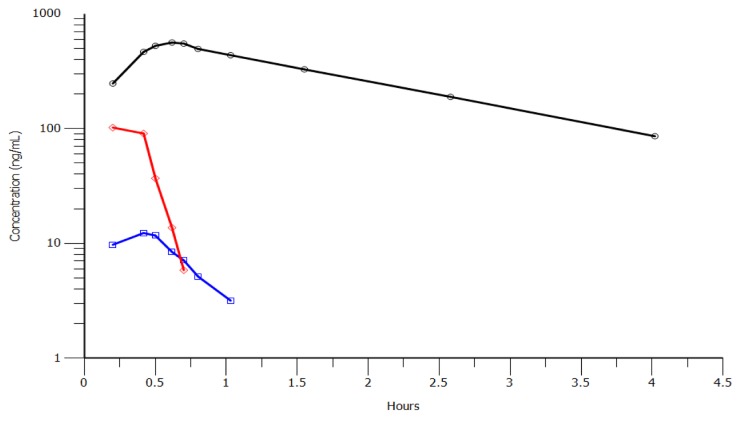
Concentration-time profiles for melflufen (red), melphalan (black) and des-ethyl-melflufen (blue) after infusion of melflufen over 30 minutes in one patient at the dose level 25 mg A very similar PK profile was obtained in dogs (not shown).

The degradation of melphalan in plasma is caused by non-enzymatic hydrolysis [24], and small peptides (similar to melflufen) are likely to have very rapid disappearance in blood from both humans and rodents [25]. Clinical PK data are available from two clinical trials in patients with solid tumors and multiple myeloma, respectively [26, 27]. During administration of melflufen as an intravenous infusion over 30 minutes, melflufen concentrations in plasma reach an early plateau or start to decrease during the later part of the infusion reflecting a rapid distribution of melflufen to cells out of the plasma compartment. After end of infusion, the half-life for melflufen decrease in plasma ranges from 1.4 to 4.9 minutes [26]. The plasma concentration of melphalan reaches levels higher than those of melflufen within 15 minutes of melflufen infusion. After end of melflufen infusion, melphalan plasma concentrations continue to increase for up to 10 minutes. This delay in peak plasma concentrations of melphalan is compatible with an extensive formation of melphalan from melflufen in peripheral tissues outside of the plasma compartment with subsequent distribution of melphalan back to blood plasma. Estimated melphalan clearance after administration of melflufen is of the same magnitude as in published studies with direct administration of equimolar doses of melphalan [28], indicating a close to complete conversion of melflufen to melphalan. The metabolite des-ethyl-melflufen reaches only very low concentrations in plasma and is eliminated with a half-life of approximately 15 minutes. Representative concentration-time profile for the compounds in one patient is shown in Figure 3. Similar profiles have been observed in the toxicological studies of dogs.

## MELFLUFEN ACTIVITY IN CELL LINES AND PRIMARY CULTURES OF TUMOR CELLS FROM PATIENTS

### Hematological malignancies

The very first publication of melflufen showed *in vitro* cellular effects of the drug in comparison with melphalan and P2, one of six alkylating peptides constituting Peptichemio, a chemotherapy cocktail synthesized by the Italian company Istituto Sieroterapico Milanese [29]. Specifically, the study showed superiority of melflufen over melphalan regarding cytotoxic activity against human tumor cell lines and primary cultures of human tumors, as evidenced by leucine and thymidine incorporation, initiation of apoptosis, and inhibition of cellular respiration. In particular, 15 primary cultures from patients with hematological malignancies were analyzed (five acute lymphocytic leukemias, three acute myelocytic leukemias, two chronic lymphocytic leukemias, and five non-Hodgkin’s lymphomas), and the results showed a mean IC_50_ value of 55 nM for melflufen, which was 27-fold lower than that of melphalan [16]. Enzymatically driven potentiation of melflufen’s activity [18] was also confirmed and compared using the lymphoma cell line U-937 *versus* the T-cell leukemia cell line CCRF-CEM. The low IC_50_-values obtained in these cell lines (0.44 and 0.13 µM, respectively) was shown to be dependent on aminopeptidase-mediated cleavage, since bestatin pretreatment markedly decreased the activity [18]. Measurements of intracellular melphalan in U-937 cells after melflufen exposure showed peak concentrations at 15 min reaching > 10-fold those obtained after melphalan exposure. In lymphoma, melflufen showed activity with cytotoxic IC_50_ values in the submicromolar range (0.011-0.92 µM) in various cell lines, corresponding to a mean of 49-fold superiority (*p* < 0.001) in potency *vs*. melphalan. In the primary cultures melflufen yielded even lower IC_50_ values (2.7 nM to 0.55 µM) and an increased ratio *vs*. melphalan (range 13-455, average 108, *p* < 0.001) [30].

Based on the indisputable value of melphalan in the treatment of multiple myeloma (MM) [31], the effects of melflufen were therefore investigated in this diagnosis, as summarized in Table 1. The resistance-based cell line panel used by Gullbo *et al.* contained three MM cell lines, RPMI-8226 and its melphalan-resistant subline 8226/LR5 and the doxorubicin-resistant cell line 8226Dox40. Melflufen was tenfold more active in all three cell lines regardless of resistance mechanism in the cell line, as expected; however, the LR5 cell line expressing high glutathione levels was slightly less sensitive (approx. 2.5-fold) [16]. Another study performed at Harvard University by Chauhan and co-workers, confirmed aminopeptidase activity in MM cells, and APN-dependent cleavage of melflufen [20]. Melflufen exposure resulted in rapid and higher intracellular accumulation of melphalan (approx. 50-fold increased exposure) and lower IC_50_-values compared to controls treated with melphalan. The *in vitro* findings were confirmed in a human MM xenograft model, showing better inhibition of tumor growth and prolonged survival of melflufen *vs* melphalan [20]. Importantly, melflufen induced apoptosis even in melphalan- and bortezomib-resistant MM cells, and acted in synergy with standard of care myeloma therapies [20]. Interestingly, melflufen triggered cytotoxicity even in p53-null ARP-1 multiple myeloma cells, suggesting that functional p53 may not be obligatory for efficient induction of melflufen-induced apoptosis. In addition, melflufen also inhibited VEGF-dependent myeloma cell migration, and tumor-associated angiogenesis, suggesting that melflufen may negatively regulate homing of myeloma cells to the bone marrow [20]. Melflufen-induced apoptosis in MM is associated with DNA damage and repair pathways, evidenced by rapid induction of gamma-H2AX, ATR, and CHK1, also in melphalan-resistant cells. Repair of the drug-induced damage is an important resistance mechanism for alkylating agents. In this aspect it is highly interesting that melphalan, but not melflufen, upregulates Ku80, which repairs DNA double-strand breaks in MM cells. Taken together, the data suggest that melflufen triggers rapid, robust, and irreversible DNA damage, which may account for its ability to overcome melphalan-resistance in MM cells [22]. Melflufen at concentrations corresponding to IC_50_ in MM cells from patients (0.1-0.5 µM) does not affect the viability of normal peripheral blood mononuclear cells [20]. Preliminary data suggest that these cells are at least 10-fold less sensitive (IC_50_ > 7µM, data presented at European Hematology Association annual meeting in Stockholm 2013).

**Table 1:** Activity of melflufen in various in vitro models of MM.

Cell designation	Cell line characteristics and reference	Sensitivity* to melflufen IC_50_ µM and reference
RPMI-8226	Sensitive maternal line (Moore 1968) Melphalan IC_50_ > 10 µM (Chauhan, Ray et al. 2013)	1.0 [16] 1.6 [20]
8226LR5	Subline of RPMI-8226, resistant to melphalan (Bellamy 1991) Melphalan IC_50_ > 10 µM (Chauhan, Ray et al. 2013)	2.6 [16] 4.5 [20]
8226Dox40	Subline of RPMI-8226, resistant to doxorubicin, mitoxantrone, acronycine, etoposide, and vincristine Sensitive to melphalan and dexamethasone (Dalton 1986)	1.8 [16] <0.5 [20]
INA-6	Dependent on IL-6 for growth (Burger 1998)	<0.5 [20]
ARP-1	Sensitive to dexamethasone	1.7 [20]
MM.1S	Sensitive to dexamethasone (Goldman-Leikin 1989, Moalli 1992)	<0.5 [20]
MM.1R	Resistant to dexamethasone (Goldman-Leikin 1989, Moalli 1992)	<0.5[20]
ANBL-6.WT	Bortezomib sensitive Melphalan IC_50_ 5.2 µM (Chauhan, Ray et al. 2013)	0.41 [20]
ANBL-6.BR	Bortezomib resistant Melphalan IC_50_ >10 µM (Chauhan, Ray et al. 2013)	0.81[20]
Primary cultures of human myeloma cells	Melphalan IC50 10 µM (Wickstrom, Haglund et al. 2008)	0.2 µM [32]

*The sensitivity has been estimated with fluorometric microculture cytotoxicity assay (Gullbo 2003 [16] and Wickstrom 2008 [32]) and 3-(4,5-dimethylthiazol-2-yl)-2,5-diphenyltetrazolium bromide (MTT) assay (Chauhan 2013 [20]). Survival-concentration bar graphs in Chauhan 2013 were re-analyzed to get the IC50-values presented.

**Figure 4:**
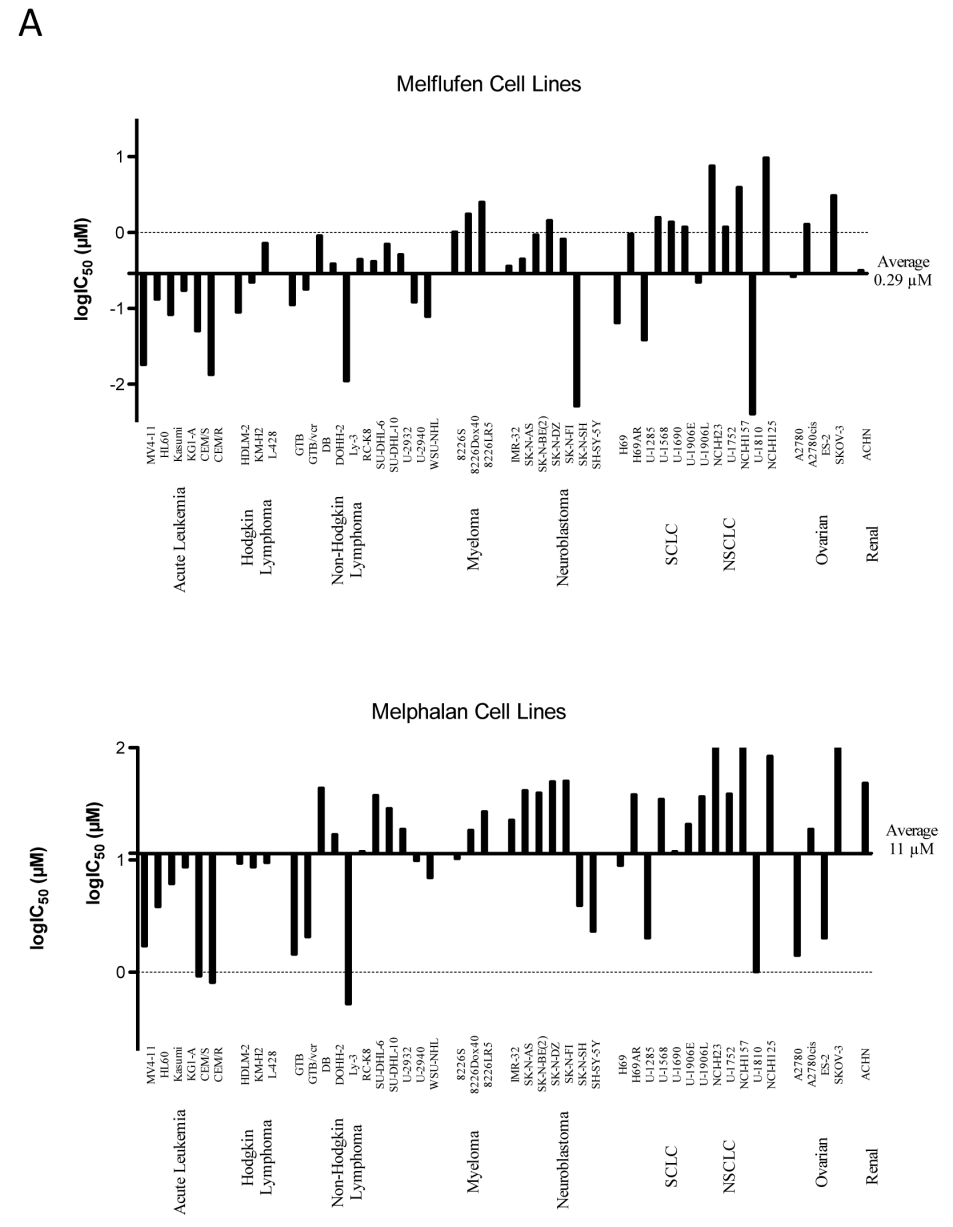

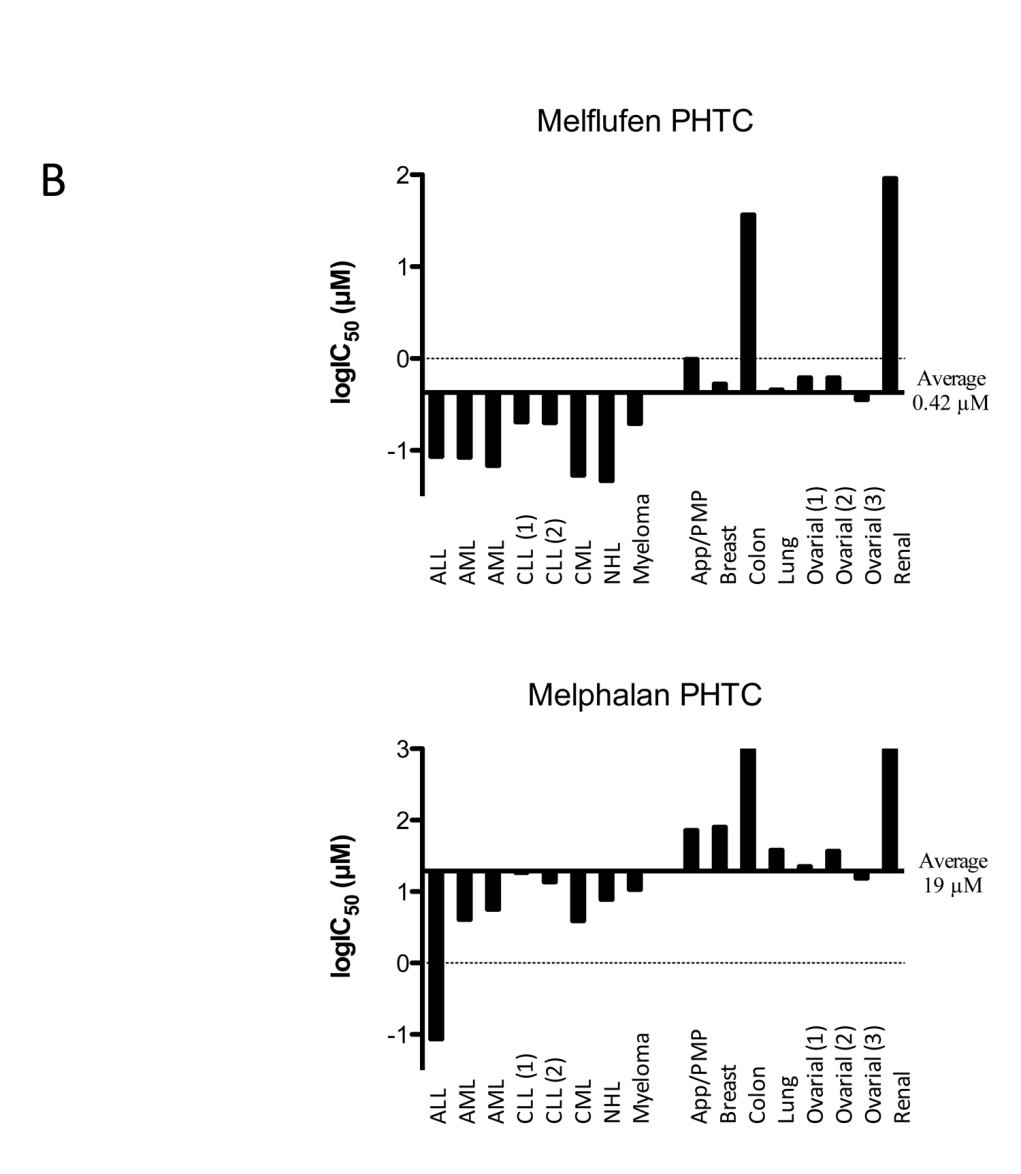
Differential activity of melflufen and melphalan in human tumor cell lines A. or primary cultures of human tumor cells (PHTCs) from patients B See text for definitions of abbreviations (tumor types). Data adapted from published results in references [17] [21] [33] [35] [41] [30] [23]. Fluorometric microculture cytotoxicity assay was used in all determinations of IC_50_. PHTC: Primary cultures of human tumor cells

A summary of results obtained with melflufen on cell lines from human malignancies is shown as a delta graph in Figure 4A. The average IC_50_ of melflufen in the 23 cell lines derived from hematological cells (acute leukemia, lymphoma, myeloma) was 0.20 µM compared to 6.9 µM for melphalan, a 35-fold improvement (Figure 4A, references in figure legend).

In the study by Wickstrom et al., the *in vitro* activity of melflufen was investigated in a large panel of primary cultures of patient tumor samples (*n* = 176) from patients with the following diagnoses: acute lymphocytic leukemia (ALL; *n* = 21), acute myelocytic leukemia (AML; *n* = 26), chronic lymphocytic leukemia (CLL; *n* = 18), chronic myelocytic leukemia (CML; *n* = 8), non-Hodgkin’s lymphoma (NHL; *n* = 14), and multiple myeloma (MM; *n* = 3). Results demonstrated IC_50_ values of melflufen in the submicromolar range (average approx. 0.1 µM). NHL was the most sensitive diagnosis *in vitro* followed by CML and AML, the latter two being more sensitive than their lymphocytic counterparts CLL and ALL. In these samples, the IC_50_ ratio of melphalan and melflufen was 50- to 100-fold [32]. A summary of results obtained with melflufen on primary cultures of patient tumor cells is shown as a delta graph in Figure 4B.

### Solid tumor malignancies

The *in vitro* studies of melflufen in various cell lines representing solid tumors showed variable efficacy. For example, the small-cell lung cancer (SCLC) cell line NCI-H69 (IC_50_ 64 nM, [17]) appeared highly sensitive compared to the non-small-cell lung cancer (NSCLC) cell line NCI-H23 (IC_50_ 7.6 µM [33]). A summary of all cell line data published so far is presented in Figure 3A. The average IC_50_ of melflufen in the 24 cell lines derived from solid tumor cells (neuroblastoma, lung cancer, ovarian cancer, and renal cell cancer) was 0.41 µM compared to 18 µM for melphalan, a 44-fold improvement.

A subsequent study was performed investigating the *in vitro* activity in seven neuroblastoma cell lines with variable drug-resistance characteristics [21]. A significantly higher potency of melflufen compared to melphalan was noted (on average 270-fold, range 85- to 810-fold). Again, the aminopeptidase inhibitor bestatin blocked melflufen- but not melphalan-induced cytotoxicity. Furthermore, melflufen-induced cytotoxicity was associated with caspase-3 cleavage and apoptotic morphology. Combination of melflufen with standard agents triggered additive or synergistic cytotoxicity even in drug-resistant cell lines (see below). Finally, melflufen efficacy was noted in xenografted mice as well (see below).

In urothelial cancer cell lines J82, RT4, TCCsup, and 5637, melflufen amplified the intracellular loading of melphalan in part *via* aminopeptidase N, resulting in increased cytotoxicity compared to melphalan alone. Melflufen induced apoptosis, seen as activation of Bak/Bax, cleavage of caspase-9/PARP-1, and induction of apoptotic cell nuclei morphology [34].

A total of 86 primary cultures of solid tumor samples from patients were investigated for melflufen-induced cell-growth inhibition in a phase II *ex vivo* study, including breast cancer (*n* = 20), colorectal cancer (*n* = 11), NSCLC (*n* = 5), ovarian cancer (*n* = 21), renal cancer (*n* = 7) and appendix cancer/pseudomyxoma peritonei (app/PMP; *n* = 22) [32]. Among these, breast cancer, ovarian cancer, and NSCLC samples were most sensitive (IC_50_ values approx. 0.5 µM), while renal and colon cancers were relatively resistant to melflufen *in vitro* (IC_50_ > 10 µM). The difference *vs*. melphalan activity was 150-fold for breast cancer samples, and approximately 75-fold for ovarian cancer, NSCLC, and app/PMP. An interesting feature of particularly high sensitivity among breast cancers with a clinically aggressive phenotype was noticed (difference *vs*. melphalan was > 700-fold) [32]. A summary of results obtained with melflufen on primary cultures of patient tumor cells is shown as a delta graph in Figure 4B.

## MELFLUFEN IN COMBINATION WITH STANDARD DRUGS

The activity of melflufen in combination with standard drugs, representing different mechanistic classes, has been studied in different human tumor cell lines [35], including neuroblastoma cell lines [21] and MM cell lines [20]. Table 2 summarizes these data, showing some potentially additive and synergistic interactions, most striking for etoposide with significant synergism in all cell lines tested (including drug resistant neuroblastoma cell line) [21].

**Table 2:** Combination analysis of melflufen and a set of standard drugs in different cell lines.

Combination Melflufen +	*CCRF-CEM*^1)^ *Leukemia*	*U937*^1)^ *Lymphoma*	*RPMI 8226*^1)^ *Myeloma*	*RPMI 8226LR5*^2)^ *Myeloma*	*MM.1S*^2)^ *Myeloma*	*ACHN*^1)^ *Renal ca*	*MCF-7*^1)^ *Breast ca*	*SK-N-AS*^3)^ *Neuroblastoma*	*SK-N-BE(2)* ^3)^ *Neuroblastoma*	*SH-SY5Y*^3)^ *Neuroblastoma*
Doxorubicin	0.8-1.2	0.8-1.2	<0.8	-	-	0.8-1.2	<0.8	0.86	0.88	0.78
Docetaxel	>1.2	0.8-1.2	>1.2	-	-	<0.8	<0.8	-	-	-
Vincristin	0.8-1.2	0.8-1.2	>1.2	-	-	0.8-1.2	<0.8	1.4	1.2	0.78
Etoposide	<0.8	<0.8	<0.8	-	-	<0.8	<0.8	0.47	0.75	0.63
Cisplatin	0.8-1.2	0.8-1.2	0.8-1.2	-	-	0.8-1.2	0.8-1.2	-	-	-
Carboplatin								0.69	1.1	0.96
5-fluorouracil	0.8-1.2	0.8-1.2	0.8-1.2	-	-	0.8-1.2	0.8-1.2	-	-	-
Bortezomib	0.8-1.2	0.8-1.2	0.8-1.2^1)^	0.091-0.914	0.512-0.913	0.8-1.2	<0.8	-	-	-
Lenalidomide	-	-	-	0.24-0.92	0.002-0.062	-	-	-	-	-
Prednisolone	0.8-1.2	0.8-1.2	0.8-1.2	-	-	0.8-1.2	0.8-1.2	-	-	-
Dexamethasone	-	-	-	0.54-0.90	0.001-0.209	-	-	-	-	-

A Combination index <1 indicates synergy, >1 antagonism. The interval 0.8-1.2 has arbitrarily been set to additive effects. All references have used the median effect method by Chou-Talalay for interaction analysis *in vitro*.

Data obtained from

1) [32]

2) [20]

3) [21]

In urothelial carcinoma cell line J82, melflufen in combination with cisplatin or gemcitabine in J82 cells resulted in additive cytotoxic effects, and for gemcitabine, also increased apoptosis induction. Profiling of melflufen-induced kinome alterations in J82 cells revealed that melflufen alone did not inhibit Src phosphorylation. Accordingly, the Src inhibitor dasatinib sensitized for melflufen cytotoxicity [34].

Using a MM cell line model, Chauhan and co-workers showed that combining melflufen with the myeloma drugs lenalidomide, bortezomib, or dexamethasone triggered synergistic anti-myeloma activity [20].

## MELFLUFEN AND ANGIOGENESIS

The role of APN in angiogenesis is well established, and the expression of the enzyme on endothelial cells is limited to angiogenic, but not normal, vasculature [7]. The expression levels of APN in primary endothelial cells and human tumor xenografts are upregulated in response to hypoxia, angiogenic growth factors, and signals regulating capillary tube formation during angiogenesis [36]. Conversely, capillary network formation is significantly inhibited by treatment with inhibitory anti-APN monoclonal antibodies or functional inhibitors [36].

**Figure 5:**
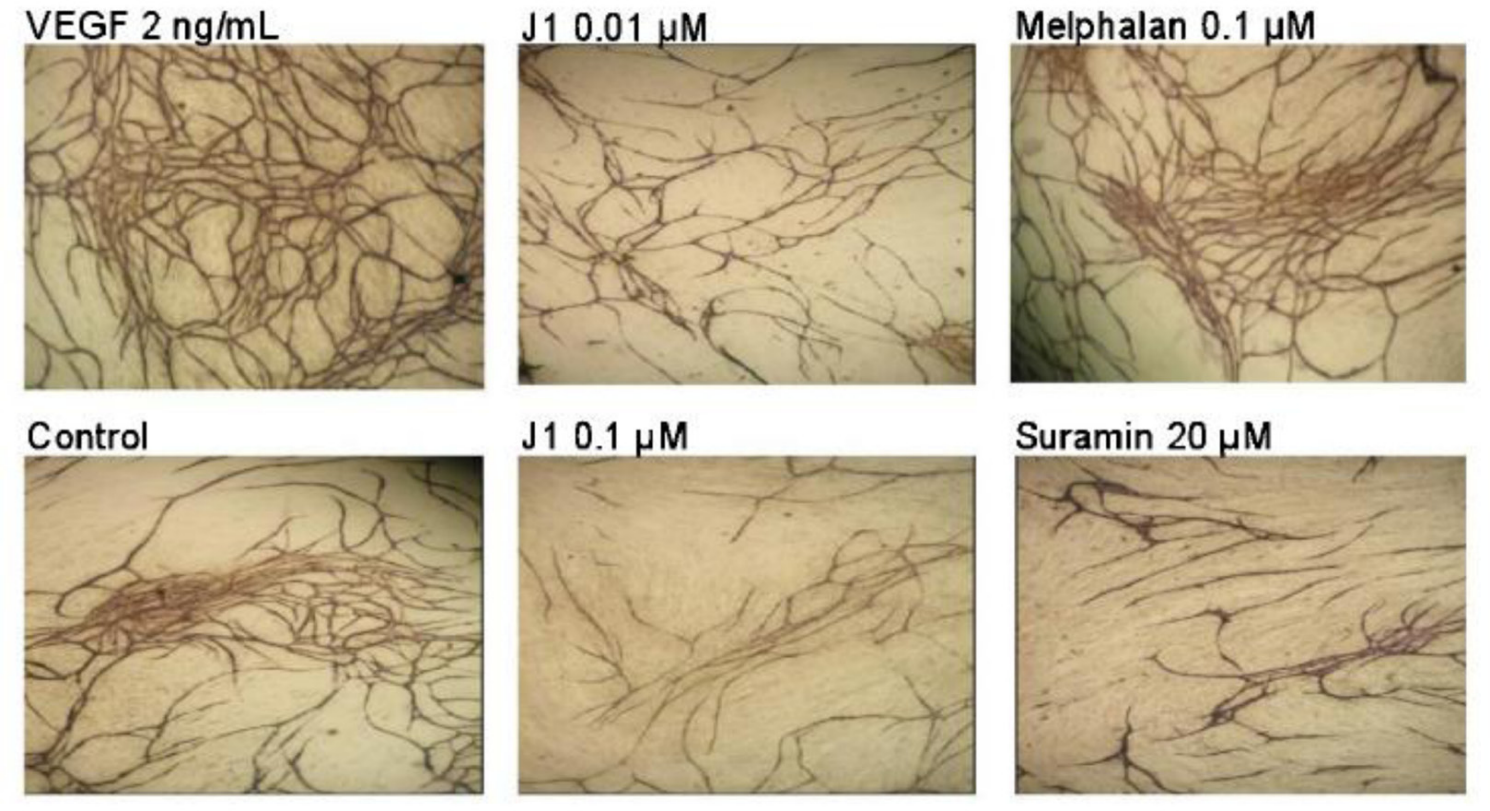
Antiangiogenic effects of melflufen (J1) in TCS Cellworks AngioKit with human endothelial cells (stained for CD31) co-cultured with fibroblasts Reprinted from Biochem Pharmacol [37], with permission.

Given the role of APN in the intracellular accumulation of melflufen, potentiating the cytotoxic effects, studies were conducted to investigate whether melflufen exhibits anti-angiogenic properties. Immunohistochemical analysis of tumors from xenografted animals demonstrated a significant decrease in number of blood vessels after melflufen treatment [21]. Anti-angiogenic properties were confirmed in a dedicated study using the chicken embryo chorioallantoic membrane (CAM) assay, and microtiter plate-based assays with human endothelial cells co-cultured with fibroblasts [37]. In concert with these observations, melflufen inhibited tubule formation in HUVEC cells by Matrigel capillary-like tube structure formation assays [20]. Finally, melflufen exhibited anti-angiogenic activity, even higher than bevacizumab, in a commercially available *in vivo* assay in mice (Cultrex DIVAA angioreactor assay) [37]. The anti-angiogenic properties of melflufen were thus pronounced and occurred in considerably lower (in some assays more than 100-fold) doses compared with those resulting in cytotoxicity. In contrast, melphalan did not show any anti-angiogenic effect at relevant concentrations (up to 10 µM), in support of the hypothesis of APN-dependent mechanism of action for this effect. Interestingly, neither melflufen nor melphalan was able to inhibit the enzymatic activity of APN at the anti-angiogenic concentrations, suggesting that APN inhibition is not the primary anti-angiogenic mechanism [37] (Figure 5).

## MELFLUFEN IN *IN VIVO* TUMOR MODELS

The *in vivo* activity of melflufen has been investigated in rodents carrying subcutaneous hollow fibers filled with human tumor cells and conventional xenograft models, as summarized in Table 3. Many of the studies have used melphalan as a positive control, and melflufen have compared favorably in a majority as indicated in the table.

**Table 3:** Summary of preclinical *in vivo* experience with melflufen in rodents

Cells	Model	Species	Dosing	Significant result for Melflufen	Reference
CCRF-CEM T-cell leukemia cell line	Hollow fiber (5-day study)	Male Sprague-Dawley rats	Single 1.33 µmol/kg	Yes (vs control and Melphalan)	[23]
RPMI8226 Multiple myeloma cell line	Hollow fiber (5-day study)	Male Sprague-Dawley rats	Single IV 1.33 µmol/kg	None (non-significant 28% reduction J1 vs. control)	
CCRF-CEM T-cell leukemia cell line	Hollow fiber (5-day study)	Male NMRI albino mice	IV 6.25 µmol/kg Q1Dx4 or Single IV 25 µmol/kg	Yes (vs control and Melphalan) Yes (vs control and Melphalan)	[38]
NCI-H69 Small-cell lung cancer cell line	Hollow fiber (5-day study)	Male NMRI albino mice	IV 6.25 µmol/kg Q1Dx4	Yes (vs control and Melphalan)	
ACHN renal adenocarci-noma cell line	Hollow fiber (5-day study)	Male NMRI albino mice	IV 6.25 µmol/kg Q1Dx4	Yes (vs control)	
CLL primary patient cells	Hollow fiber (5-day study)	Male NMRI albino mice	Single IV 25 µmol/kg	None (non-significant 70% reduction J1 vs. control)	
Ovarian carcinoma primary cancer cells	Hollow fiber (5-day study)	Male NMRI albino mice	Single IV 25 µmol/kg	Yes (vs control)	
SK-N-BE(2) Neuroblast-oma cell line	Subcutaneous xenograft	Male nude rats (HsdHan: RNU-rnu; Harlan)	Single IV 10 µmol/kg	Yes (vs control)	[21]
SH-SY5Y Neuroblastoma	Subcutaneous xenograft	Female nude mice (NMRI nu/nu)	IV 0.50 µmol/kg day 0 and 6	Yes (vs control and Melphalan)	
MM.1S Multiple myeloma	Subcutaneous xenograft	Male triple immune-deficient BNX mice	IV 3 mg/kg Q2Wx2W	Yes (vs control and Melphalan)	[20]
Multiple myeloma	Genetically Engineered Mouse Model	Immunocompetent Vk*MYC Mice	IP 4 mg/kg 2QWx3W	Yes (vs control, numerically better than melphalan but no statistics)	[39]
A2780 Ovarian carcinoma	Subcutaneous xenograft	Female SCID mice	IV 4-8 mg/kg 2QWx3W	Yes (vs control and Melphalan)	[41]
SK-OV-Luc Ovarian carcinoma	Intraperitoneal or subperitoneal xenograft	Female athymic nude-foxn1nu mice	IP 4 mg/kg 3QWx2W	Yes (vs control)	
DOHH lymphoma cell line	Subcutaneous xenograft	Female C.B-17 Scid mice	IV 3 mg/kg 2QWx2W	Yes (vs control)	[30]
Pdx (FAB M1) AML	Patient derived AML	Female SCID mice	IV 5-8 mg/kg 2QWx2W or single dose 16 mg/kg	Yes (vs control and Melphalan)	[23]

In studies with direct comparison of melflufen vs. melphalan the effect was compared at equimolar doses (no study has shown melphalan superiority). Melphalan treatment was not evaluated in SK-OV-Luc ovarian carcinoma and DOHH Lymphoma experiments.

To determine a safe and tolerable dose for the efficacy studies, a dose-ranging study in mice was conducted. Specifically, two or four intravenous injections (tail vein) of melflufen or melphalan were administered over 14 days, and the effects on the weight gain and hematological parameters were monitored. The highest tested dose of 25 μmol/kg (13 mg/kg; corresponding to approx. 48 mg/m^2^), was considered tolerable, with minor effects on weight gain but a significant effect on white blood cell counts [38]. Interestingly, melphalan and melflufen showed comparable toxicity (animal weight and blood cell counts) based on the molar dose [38], a feature that has been observed also in subsequent efficacy studies [20, 21, 38, 39].

The hollow fiber model in mice [40] was used to investigate the activity of four daily doses of melflufen (total 25 µmol/kg) against three cell lines (the T-cell leukemia CCRF-CEM, the small-cell lung cancer NCI-H69, and the renal adenocarcinoma ACHN), and a single identical dose against two samples of patient tumor cells (CLL and ovarian carcinoma, respectively). A significant melflufen activity was noted in four of the five cell lines tested (all but CLL) [38]. In addition, significant activity of melflufen in mice models has been observed in lymphoma [30] and AML [23]. An intra- and subperitoneal xenograft model showed activity of intraperitoneal administered melflufen for peritoneal carcinomatosis, with minimal side effects and modest systemic exposure [41].

Very low dose melflufen treatment (single dose of 0.5 µmol/kg) was found to be effective in nude mice xenografted with SH-SY5Y neuroblastoma tumor cells. Moreover, immunohistochemistry of tumor sections from melflufen- but not melphalan- or vehicle-treated mice showed increased caspase-3 activation, reduced Ki67 positivity, and decreased mean vascular density, thus suggesting apoptosis and anti-angiogenic efficacy [21]. This low dose was similarly effective in nude rats xenografted with the same cells (SH-SY5Y). A higher, but tolerable, dose (10 µmol/kg) of melflufen was effective in nude rats xenografted with the resistant SK-N-BE(2) cell line, showing significant tumor growth inhibition, apoptosis, and decreased proliferation [21].

The *in vivo* effects of melflufen in a multiple myeloma model were examined using SCID mice xenografted with myeloma cells (MM-1S), using a repeated dose schedule (3 mg/kg twice a week for two weeks, corresponding to 5.5 µmol/kg). A significant delay in tumor growth and prolongation of survival was observed in mice receiving melflufen (median survival: untreated = 24 days; melflufen = 44 days) [20].

Rodent models with xenografted human tumor cell lines have been criticized for poor predictability of clinical outcome. In this context, the Vk*MYC transgenic mouse with spontaneously occurring myeloma tumors has been suggested as an alternative model to predict single-agent drug activity [39]. Interestingly, melflufen in this model (given as 4 mg/kg i.p. injection twice weekly) was highly effective, showing a Vk*MYC response (defined as > 50% reduction in M-spike at 14 days) in 66% of treated animals, and the average M-spike reduction ranked highest out of 18 investigational anti-multiple myeloma agents examined [39].

In conclusion, the *in vivo* antitumor activity of melflufen has been shown in 14 different models. Direct comparison with melphalan has been done in at least 7 separate experiments, repeatedly showing the superiority of melflufen at equimolar doses without signs of overt toxicity.

## CLINICAL EXPERIENCE WITH MELFLUFEN

In a combined phase I/IIA study in patients with late-stage solid tumor malignancies intravenous melflufen was administered on a Q3W schedule. In conclusion, this study showed that melflufen can safely be given to cancer patients, and the toxicity profile was as expected for alkylating agents; the recommended phase 2 dose was identified as 50 mg Q3W. Reversible and manageable bone marrow suppression was identified as dose-limiting toxicity, preferentially in heavily pretreated patients. Clinical activity was suggested in ovarian cancer, but modest activity in treatment of refractory non-small-cell lung cancer [26].

Final data from the phase I/II study with melflufen in combination with 40 mg weekly dexamethasone in patients with relapsed and relapsed-refractory multiple myeloma (RRMM) (NCT01897714) were presented at the annual European Hematology Association (EHA) congress in Copenhagen, Denmark, in June 2016 [27]. The phase I part of the study showed that 40 mg of melflufen could safety be given monthly in combination with weekly 40 mg dexamethasone as the maximal tolerated dose (MTD). The final phase II data in 40 patients with median 4 (2-9) prior lines of anti-myeloma therapy treated with MTD were presented. Melflufen has promising activity in heavily pre-treated RRMM patients where conventional therapies have failed, with an overall response rate (partial response [PR] or better) of 40% and clinical benefit rate (minimal response [MR] or better) of 63% in efficacy-evaluable patients using the International Myeloma Working Group Criteria [42]. Similar results were seen across patient populations regardless of refractory status. It was of special interest that 53% of the alkylator refractory patients responded to melflufen treatment with a PR or better and 73% with a MR or better. The median progression-free survival (PFS-50%) was 4.3 months (95% CI: 3.7 to 8.5), and the PFS-25% was 9.7 months (95% CI: 7.9 to 14) based on 37 events in all 40 treated patients. Seventeen patients (43%) were progression-free at 6 months and 5 patients (12.5%) at 12 months. The median duration of response (DOR) was 7.7 months (95% CI: 4.6 to ∞) based on 11 events in 12 responding (≥PR) patients. While non-hematologic adverse events were infrequent, hematologic toxicity was common, but manageable, with cycle prolongations, dose modifications, and supportive therapy.

## DISCUSSION

Aminopeptidases are widely distributed enzymes catalyzing the cleavage of amino acids from the amino terminus of protein or peptide substrates, and may localize as subcellular organelles in cytoplasm or as membrane components. Along with other hydrolytic enzymes, several aminopeptidases have been described as being overexpressed in human malignancies, suggesting their utilization as anti-tumor targets [1]. Among the aminopeptidases, the multifunctional protein APN has by far received the most attention due to its association with the phenotypes of human malignancies (e.g. cell proliferation, secretion, invasion, and angiogenesis) [3, 4, 11, 12].

Melphalan is a well-known cytotoxic chemotherapy used clinically since the 1950-ties. Melflufen is a targeted, peptidase-potentiated chemotherapeutic agent. The mechanism of action of melflufen in combination with the high peptidase activity in tumor cells, results in 50-100 fold higher intracellular concentration of alkylating moieties inside tumors cells following melflufen treatment compared with equimolar concentrations of melphalan in cell culture experiments. Further experiments in myeloma cells have shown additional antitumor mechanisms including anti-angiogenic properties and reduced DNA repair. Melflufen has shown statistically significant antitumor superiority over equimolar melphalan in 7 tumor-bearing animal models without signs of increased toxicity. Early clinical data suggest clinical activity in advanced relapsed and refractory multiple myeloma patients regardless of refractory status. The efficacy has been preserved also in alkylator refractory patients.

Melflufen, which is very lipophilic, readily enters cells. Through the action of peptidases such as APN, the peptide bond of the molecule is cleaved, resulting in intracellular release of melphalan, which due to its hydrophilicity is trapped inside the cell. The rapid continuous inflow of melflufen is driven by a rapid intracellular clearance of the drug by APN-mediated hydrolysis. Despite identical alkylating groups in melphalan and melflufen, numerous *in vitro* and/or *in vivo* studies have documented a significantly higher cytotoxic potency of melflufen. Through peptidase potentiation, melflufen yields high intracellular concentrations of melphalan, resulting in extensive DNA damage, apoptosis, and cell death as well as anti-angiogenic effects, as evidenced by considerably lower IC_50_ values of melflufen compared to melphalan also in drug-resistant cells. It is hypothesized that melflufen could provide better efficacy but no more toxicity than what is achieved with melphalan, an assumption so far supported by several animal studies and early data from clinical trials.

Non-clinical safety studies conducted to support clinical studies in relapsed and refractory MM patients have consisted of safety pharmacology studies in rats (CNS, respiratory and cardiovascular endpoints), single dose toxicity studies in mice, rats and dogs [26]. The toxicity pattern observed in mouse and rat repeat dose studies was consistent with that observed with melphalan i.e. effects on the white blood cell lineage and histological changes seen primarily in lung, testes and lymphoid organs. In the dog, the spectrum of toxicity was quite similar to that in mice and rats. These studies gave thus no indication of a different toxicity spectrum of melflufen compared to that of melphalan

Among diagnoses studied to date, melflufen show significant higher activity than melphalan in neuroblastoma [21], lymphoma [30], AML [23], and multiple myeloma models [20]. Furthermore, combination experiments of melflufen and standard drugs used to treat these diagnoses reveal several examples of synergy, for example, with etoposide or lenalidomide. *Ex vivo* screens of primary cultures from patients do, not surprisingly, suggest hematological malignancies as target indications, with very low IC_50_ values for both chronic and acute leukemias as well as lymphomas and myelomas [32]. Interestingly, melflufen is more active against myeloid than lymphoid leukemias (evidenced by IC_50_, and as a ratio of melphalan/melflufen). These findings are consistent with the identification of APN as a pro-apoptotic target in AML cells [43]. In solid tumors, the activity of melflufen is generally lower, but the IC_50_ values obtained in primary cultures of breast, ovarian, and NSCLC cancers are still in the submicromolar range, and some samples from patients with locally advanced or clinically aggressive breast cancer showed very high sensitivity to the drug [32].

Melflufen is comparably stable in cell growth medium with a degradation half-life of approximately 2 hours [33], and a similar stability in plasma is expected. In humans, whole blood degradation is most likely more rapid suggested from the experience with similar peptides [25], and the short half-life noted in patients from the clinical trials. From *in vitro* experience, it is clear that a comparably short exposure of melflufen, approx. 30 minutes, is sufficient to yield a maximal effect in tumor cells [18]. During a 30-min infusion of melflufen, an equilibrium concentration is built up, and while melflufen is distributed to cells, melphalan is preferentially accumulated in those with high expression of aminopeptidases like APN. In human cancer subjects, melflufen is rapidly distributed from the blood stream to cells, a steady-state concentration is reached during the infusion (30 minutes) [26]. After the infusion stops, melflufen concentration declines with a half-life of 2.8 minutes (range 1.4 - 4.9). The formation of melphalan, on the other hand, continues and by redistribution Cmax for melphalan is obtained shortly after the infusion is stopped. The only possible explanation is a very rapid distribution of intact melflufen to peripheral tissues outside of the plasma compartment, contributing to the disappearance from plasma. The preferential trapping of melphalan in APN-expressing cells are hypothesized to contribute to improved therapeutic index of melflufen demonstrated in several experimental *in vivo* models of human cancer.

Phase I/II clinical trials of melflufen in more than 125 patients with solid tumors and relapsed and refractory multiple myeloma (RRMM) showed that 40 mg of the drug can safely be given on a monthly schedule in combination with weekly 40 mg dexamethasone and that dose is limited by dose-dependent, reversible, monitorable, and mechanism-driven hematological toxicity [26, 27]. The clinical results in RRMM have been promising with approximately 40% of the patients responding with a partial response or better in efficacy evaluable patients.

Following discussions with relevant Regulatory Agencies, melflufen has been approved for phase 3 studies in relapsed and refractory multiple myeloma. The phase 3 studies are expected to start during 2017. Future possibilities include the use of melflufen for conditioning prior to autologous stem cell transplantation, treatment of amyloid light-chain (AL) amyloidosis and treatment of other hematological malignancies.
